# Therapeutic advances and open questions in postpartum-depression research

**DOI:** 10.1016/j.ebiom.2023.104925

**Published:** 2023-12-13

**Authors:** eBioMedicine

On August 4, 2023, the US Food and Drug Administration (FDA) approved zuranolone for postpartum depression. In a comprehensive definition that encompasses perinatal depression, postpartum depression occurs during pregnancy or within 1 year after childbirth. It is a depressive episode during which people experience feelings of sadness, guilt, and worthlessness; sleep disturbances; often increased anxiety; anhedonia; and, in its more severe form, suicidal ideation. This approval will considerably facilitate treatment access in the USA because zuranolone is the first oral medication to be approved for postpartum depression. The only previous medication approved by the US FDA specifically for postpartum depression, brexanolone, is required to be administered intravenously in a health-care facility. However, because of its high cost and uncertainties regarding health-insurance coverage, zuranolone might also widen the socioeconomic disparities in postpartum depression.

There is increasing evidence of higher rates of postpartum depression in low-income and middle-income countries than in high-income countries. A 2021 systematic review published in *Translational Psychiatry* estimated that postpartum depression affected about 17% of women who were pregnant or parturient worldwide, with substantial disparities across geographical regions. An analysis of three population-based national maternity surveys in England published in 2023 in *The Lancet Regional Health–Europe* highlighted a concerning pattern that might indicate an increasing prevalence of postpartum depression, which was exacerbated by the negative effects of the COVID-19 pandemic on perinatal mental health. Specifically, the prevalence of postpartum depression in England increased from approximately 10% in 2014 to 16% in 2018, and further increased to almost 24% in 2020.

Identifying the risk factors and mitigating factors of postpartum depression is crucial in the implementation of perinatal mental health programmes. Some well known sociodemographic factors and health conditions that are associated with postpartum depression include social support, education, partnership status, pregnancy planning, domestic violence, long-term poor mental health, antenatal anxiety, and depression. Associations between postpartum depression, difficult pregnancy-related and childbirth-related experiences, and obstetric complications have also emerged. In a US population-based retrospective cohort study published in 2023 in *eClinicalMedicine*, patients with ischaemic placental disease (IPD)—defined as a syndrome of preeclampsia, placental abruption, or small-for-gestational-age birth—during pregnancy had a higher adjusted hazard ratio for risk of readmission to hospital for postpartum depression than patients without IPD. An even greater risk was found for patients with preeclampsia with severe features or with two or three forms of IPD. Poorly studied modifiable environmental risk factors should also be considered in postpartum depression, as highlighted in a 2023 retrospective cohort study in California, USA, published in *JAMA Network Open*. The study showed slightly increased risks of postpartum depression associated with increased exposure to some air pollutants during the antepartum and postpartum periods. Risk factors might have long-term repercussions in the brain and characterising them, for example in animal models, might help reveal the mechanisms of postpartum depression. In a 2023 publication in *Nature Communications*, female mice that were exposed to social isolation—a form of psychosocial stress—during late adolescence exhibited abnormal social behaviour during the postpartum period. Using optogenetics and in-vivo calcium imaging, the study showed that the behavioural effect resulted from an hypofunctional anterior insula to prelimbic cortex pathway, leading to a reduction in normally occurring neuronal activation in the prelimbic cortex during social encounters.

As the risk factors for postpartum depression are being refined, the causes and pathophysiological mechanisms of postpartum depression are yet to be firmly established. An analysis of a cohort study published in *The Lancet Psychiatry* in 2017 suggested that postpartum depression is a heterogeneous disease with multiple symptom dimensions distinguishing several clinical phenotypes with varying time of onset. Thus, elucidating the potentially multiple underlying mechanisms is important as it might lead to recognising specific therapeutic targets, possibly helping to stratify people in clinical trials and eventually improving treatment outcomes. Whether postpartum depression is a distinct clinical entity from major depressive disorder (MDD), for example, is still debated. Meta-analyses of genome-wide association studies were published in the *American Journal of Psychiatry* in 2023. They indicated both overlap and divergence between these two forms of depression at the genetic level.

The US FDA approval of zuranolone was linked to a publication in the *American Journal of Psychiatry* of the latest phase 3, double-blind, randomised clinical trial, in which the depressive symptoms of women with severe postpartum depression that were assessed at day 15—the study’s primary outcome—were significantly improved in women receiving zuranolone 50 mg per day for 14 days compared with the depressive symptoms of women receiving placebo. Both zuranolone and brexanolone structurally mimic allopregnanolone, which is a progesterone metabolite produced by the corpus luteum and placenta during pregnancy, with substantially reduced amounts after parturition. This neuroactive steroid acts as a positive allosteric modulator of γ-aminobutyric-acid type A (GABA_A_) receptors, enhancing GABA inhibition of neuronal activity. The rapid therapeutic effects of zuranolone or brexanolone support the hypothesis of an excitation–inhibition imbalance and GABAergic signalling dysfunction in postpartum depression. But the story is certainly more complex. In a 2023 longitudinal study published in *eBioMedicine*, blood levels of inflammatory mediators, including TNF-α and IL-6, were reduced after intravenous brexanolone infusion in participants with postpartum depression. The response of these inflammatory mediators to immune activators was also inhibited. The magnitude of these effects correlated with improvement in depression scores, supporting accumulating evidence of the involvement of inflammatory pathways in postpartum depression and suggesting their contribution to the therapeutic action of brexanolone.

Hormone sensitivity, another putative pathophysiological underpinning of postpartum depression, was investigated in a cohort study of 22 years of Danish health registry data published in *JAMA Psychiatry* in 2023. Women with a history of depression associated with the initiation of hormonal contraception were more susceptible to postpartum depression than women with a history of depression that was not associated with hormonal-contraception use, implying that a subgroup of people with postpartum depression might be hormone sensitive. Additionally, the dysregulation of cellular stress signalling, probably recruited during pregnancy and parturition, might be implicated in postpartum depression according to a 2023 study published in *Molecular Psychiatry* that transcriptionally compared lymphoblastoid cell lines derived from women that had and women that had not previously experienced postpartum depression.

The multiple putative pathophysiological mechanisms of postpartum depression might lead to common brain-network alterations. Published in *Frontiers in Psychiatry* in 2022, a review of 26 neuroimaging studies assessing brain correlates of postpartum depression supported this idea as it reported consistent changes across studies, namely functional, structural, and metabolic changes in the default mode and salient networks and changes in the activity of the corticolimbic system during emotional tasks in women with postpartum depression. As for genetic evidence, some neuroimaging features of postpartum depression overlap with those of MDD, whereas others are distinctive. Neuroimaging markers of postpartum depression could be integrated into clinical trials in the future as secondary assessments of therapeutic efficacy beside symptoms alleviation.

Postpartum depression has immediate and long-term detrimental effects on health. It also adversely affects birthing parent–infant attachment and family life and the physical, behavioural, emotional, and cognitive development of a child. Despite these consequences, many questions are yet to be explored. For example, an improved understanding of the biological actions of allopregnanolone-mimicking medications that contribute to therapeutic effects lasting at least 30 days after treatment, despite their rapid clearance, is needed. Whether these medications affect the safety of chestfeeding, or the birthing parent–infant bond are other crucial unknowns. At *eBioMedicine*, we welcome translational and early-evidence clinical studies aiming to clarify the molecular, cellular, and neural substrates of postpartum depression, as research should proceed to propose alternative therapeutic interventions for people who are unresponsive to available therapies.

